# Candidate Stress-Specific microRNA Expression in Pediatric Thrombosis and Healthy Children: A Comparative Analysis of Serum miR-34a-5p in Relation to Stress Levels and Inflammatory Genetic Traits

**DOI:** 10.3390/genes17091124

**Published:** 2026-09-16

**Authors:** Iphigenia Gintoni, Elissavet Damaskopoulou, Zoi Siouti, Kleoniki Baldouni, Maria Skoufou, Nikolaos Pachis, Athina Dettoraki, Aikaterini Michalopoulou, Helen Pergantou, Dimitrios Vlachakis, George P. Chrousos, Christos Yapijakis

**Affiliations:** 1 Unit of Orofacial Genetics, 1st Department of Pediatrics, School of Medicine, National Kapodistrian University of Athens, “Aghia Sophia” Children’s Hospital, 115 27 Athens, Greece; iph.gintoni@gmail.com (I.G.); sioutizoi@yahoo.gr (Z.S.); kleoniki_km@hotmail.com (K.B.); nikopach92@yahoo.gr (N.P.); 2 Choremion Laboratory, University Research Institute of Maternal and Child Health and Precision Medicine, “Aghia Sophia” Children’s Hospital, 115 27 Athens, Greece; dimvl@aua.gr (D.V.); chrousge@med.uoa.gr (G.P.C.); 3 Laboratory of Genetics, Department of Biotechnology, School of Applied Biology and Biotechnology, Agricultural University of Athens, 118 55 Athens, Greece; edamaskopoulou@gmail.com; 4 Haemophilia Center for Children and Adolescents/Haemostasis and Thrombosis Unit, “Aghia Sophia” Children’s Hospital, 115 27 Athens, Greece; athinadett@gmail.com (A.D.); katia.michalopoulou@gmail.com (A.M.); hpergantou@gmail.com (H.P.)

**Keywords:** thrombosis in childhood, adolescent thrombosis, coagulation, thrombophilia, stress, perceived stress, miRNA, miR-34a, epigenetics, interleukin 1-beta, +3953 C/T polymorphism

## Abstract

Background: Pediatric thrombosis (PT) is a rare clinical entity manifesting from neonatal life to adolescence with possibly severe clinical complications and increasing prevalence in the last decade. Its pathogenesis has been attributed to a multitude of factors, all of which share stress and inflammation as a common denominator. Although stress-induced inflammation, marked by the upregulation of proinflammatory interleukins, such as IL-1β, creates a prothrombotic environment, its relation with thrombosis remains incompletely defined, especially in the context of PT, which differs substantially from adult thrombosis. Here, we aimed to epigenetically explore the potential link between PT and stress-induced inflammation through a combinatorial approach. Methods: Initially, we bioinformatically identified candidate stress-specific miRNAs in childhood, followed by subsequent experimental assessment in serum samples of PT patients and healthy controls in relation to subjectively perceived stress levels and the functional +3953C/T genetic polymorphism of the *IL1B* gene, which affects the circulating levels of the proinflammatory and stress-induced interleukin 1beta (IL-1β). Results: The bioinformatic analysis identified miR-34a-5p as the most stress-specific candidate microRNA in childhood, exhibiting the highest gene-target coverage (80.6%) of the developed panel of adversity-related genes, but is also known to regulate *IL1B* expression. The expression of serum miR-34a-5p was not significantly influenced by subjectively perceived stress; however, its expression was significantly reduced in the presence of the T allele of the functional +3953C/T polymorphism of its target gene, *IL1B*, associated with increased levels of the stress-induced proinflammatory and simultaneously prothrombotic IL-1β cytokine (FC:0.57; *p* = 0.024). Conclusions: The observed downregulation of miR-34a-5p in the presence of the IL-1β-increasing T allele possibly indicates a dynamic regulatory relationship, which, alongside its previously established long-term downregulation in PT, renders miR-34a-5p a promising candidate molecule for bridging the biological processes of stress-associated inflammation and thrombosis. In addition, its bioinformatically yielded candidate stress-specific role holds great potential for the non-invasive evaluation of adversity-related stress in childhood.

## 1. Introduction

Pediatric thrombosis (PT) represents a rare clinical entity that involves the pathological formation of intravascular blood clots, leading to obstructed circulation [1,2], which in turn may induce severe life-threatening clinical complications. Even after treatment, a large portion of affected children and adolescents remain at risk for long-term thrombotic sequelae, such as post-thrombotic syndrome, while around 50% exhibit persistent hypofibrinolysis, thereby leading to hemostatic dysfunction [3,4,5]. During the last decade, a predominant increase of approximately 70% has been reported in PT rates, with an incidence of up to 14 cases per 10,000 hospital admissions [1,2].

In a descending order of frequency, the main types of pediatric thrombosis include cerebral venous sinus thrombosis (CVST), accounting for around 50% of cases, followed by deep vein thrombosis (DVT) of the lower extremities, representing approximately 30%, and finally pulmonary thromboembolism, in 20% of admissions [2,5,6]. A multitude of factors can contribute to the development of pediatric thrombosis, including genetic predisposition to thrombophilia, placement of central venous catheters, malignancy, trauma, and inflammation, all of which may further challenge the already imbalanced and immature pediatric hemostasis, which is characterized by significant hypofibrinolysis. Although most of these factors might appear diverse, they all share stress as common denominator [1,7,8].

As the main manifestation of cardiovascular disease, thrombosis is known to be highly influenced by both physiological and psychological stress, which lead to a prothrombotic state of high coagulability; therefore, they are considered to be pathological triggers [8,9]. High coagulability is associated with substantial endothelial dysfunction and stress-induced inflammation, which promote thrombosis in a self-perpetuating cycle. In fact, while acute stress has been shown to enhance both coagulation and fibrinolysis, chronic stress appears to shift hemostasis toward a prothrombotic state, through the activation of coagulation and the simultaneous impairment of fibrinolytic capacity [9]. Anxiety-related disorders and depression, which are associated with the increased activation of stress-related pathways, have been shown to independently increase DVT risk, due to their influence on the autonomous nervous system (ANS) and the bold induction of systemic inflammation [10].

Although the relationship between stress-induced inflammation and thrombosis remains incompletely defined, the respective prothrombotic shift appears to be reflected through the upregulation of specific proinflammatory mediators, as both physiological and psychological stress are characterized by a significant elevation of circulating inflammatory markers. Those include interleukins (ILs), a group of cytokines that perform as the main messaging network of the immune system, by providing necessary inflammatory defenses in order to maintain systemic homeostasis [11,12,13]. Particularly, IL-1β, IL-2, IL-6 and IL-10 progressively increase following stressful events and are highly influenced by perceived psychological and socioeconomic stress [9,14].

To date, there are no available studies investigating the influence of psychological stress on the inflammatory profile of patients with cardiovascular diseases, such as thrombosis. Nevertheless, it is widely accepted that the connective tie between stress levels and the upregulation of inflammatory markers appears to be the activation of the hypothalamic–pituitary–adrenal (HPA) axis, responsible for cortisol production, and ANS [9,15,16]. Among inflammatory markers, IL-1β in particular poses as a key candidate for bridging stress to thrombosis, since its systematic proinflammatory increase induces vascular permeability and immune cell recruitment, the two pillars for thrombotic development [7,11,13,17]. Additionally, HPA axis dysregulation, induced by the exposure of children to severe stressors, leads to the reduction in glucocorticoid receptor (GR) responsiveness and cortisol rhythms. In this case, the receptors fail to counterbalance the stress-induced inflammation, thereby leading to the long-term activation of the IL-1β pathway [18,19].

In this context, pediatric thrombosis represents an even more complex and challenging field, as data derived from adults cannot be directly inferred to children, particularly given that the body of adult data is itself limited. Most importantly, pediatric thrombosis cannot be regarded as a counterpart of the adult form due to its distinct pathogenetic features that in turn reflect fundamental differences in hemostatic physiology and genetic predisposition [7]. Therefore, it is safe to assume that the relationship between stress and thrombosis may differ in pediatric populations, since, unlike adults, children are exposed to different sources of stress in daily life, while their biological stress responses may be strongly shaped by adverse childhood experiences, which may have measurable and long-lasting biological effects during development, thereby influencing cardiovascular and neuroendocrine functions, as well as inflammatory profiles.

More specifically, stressors in childhood can have a strong impact on the developing HPA axis and may result in changes in immune response [15]. These stressors, be they acute or chronic, have been shown to result in the upregulation of proinflammatory mediators, including interleukins within the body, leading to a state of chronic inflammation and the setting up of a proinflammatory feedback loop [9,15,16,20]. In fact, adverse childhood experiences may lead to upregulated expression patterns of proinflammatory factors that may persist from childhood into adulthood, ultimately increasing the risk of cardiovascular conditions, including stroke [21]. Children who have been exposed to early-life stress, and children suffering from post-traumatic stress disorder (PTSD), have been found to have elevated levels of basal and stress-induced IL-6, which is known to activate the HPA axis and is also significantly increased in patients with pediatric thrombosis [22].

Another challenge in studying the interaction between stress and thrombosis in childhood lies in the difficulty of accurately assessing both biological and psychological stress. The latter, in particular, is highly influenced by a child’s developmental stage, emotional expression and their own perception of stressful experiences. In this context, the evaluation of stress in children currently relies on a combination of subjective and objective methods, each carrying distinct advantages and limitations. Subjective approaches include self-report questionnaires that capture the perception of stress exposure and emotional symptoms, such as the Child Perceived Stress Scale (PSS-C) [23,24]. These tools are limited by age-appropriate comprehension, reliance on recall (particularly in retrospective measures), potential reporter bias when completed by parents rather than the child, and the inability to capture the physiological dimension of the stress response, in contrast to objective physiological methods, such as salivary or hair cortisol measurement, which are complicated by daily fluctuations and a lack of established reference ranges [25,26,27].

Consequently, there is currently no fully reliable method for assessing stress in childhood, whether biological or psychological. This particular limitation further challenges not only the identification of significant stress exposure but also hinders the investigation of its potential contribution in disease development. To begin unraveling that murky field, adverse childhood experiences, such as abuse, neglect, emotional deprivation, and other forms of persistent adversity, are among the most extensively studied sources of childhood stress and may provide both a targeted framework and a valuable source of stress-related data [28,29]. Growing evidence indicates that the consequences of adverse childhood experiences extend beyond psychosocial functioning and become biologically embedded through molecular mechanisms that influence physiological regulation throughout the lifespan, with epigenetic regulation gaining increasing recognition as a central linkage between environmental exposures and biological adaptation [30,31].

Among the key epigenetic mediators, microRNAs (miRNAs), a class of small non-coding RNAs that negatively regulate gene expression through the complimentary binding onto the 3’ untranslated regions (UTRs) of their mRNA targets, have gained particular attention in the context of early-life stress [32,33,34]. This is attributable not only to their ability to modulate key biological processes, such as inflammation, through the regulation of their gene targets, but also to their characteristic and measurable expression patterns across different conditions, including both stress and thrombosis [7,32].

On the other hand, within the context of pediatric thrombosis, our group has recently conducted the first worldwide study exploring miRNA involvement. Based on strategic bioinformatic predictions and subsequent experimental study, we identified miR-34a-5p, which epigenetically regulates the angiotensin-converting enzyme/plasminogen activator inhibitor-1 (ACE/PAI-1) axis that induces hypofibrinolysis in PT. Serum miR-34a-5p was significantly downregulated by approximately 40% in children with thrombotic episodes, compared to healthy controls. Its expression changed dynamically during the post-thrombotic course, in parallel with anticipated circulating ACE and PAI-1 fluctuations, thereby supporting a strong regulatory relationship, but also a long-term impaired regulatory efficiency, thereby posing as a potential marker of fibrinolytic capacity in high-risk children [7].

In the present study, we aimed to epigenetically explore the potential link between stress and pediatric thrombosis by initially identifying candidate stress-specific miRNAs in childhood through a multifactorial bioinformatic approach, leveraging on the genetic basis of early-life stress together with the available data on miRNA dysregulation in the biospecimens obtained from children exposed to adverse childhood experiences. MiRNA expression was subsequently assessed in the serum of children with thrombotic episodes and healthy controls, and were evaluated within each group, in relation to perceived stress levels and the functional +3953C/T genetic polymorphism, in exon 5 of the *IL1B* gene, which, in the presence of the T allele, is associated with increased circulating levels of the proinflammatory and stress-related IL-1β.

## 2. Materials and Methods

### 2.1. Sample Collection

The present study was bioethically approved by the Ethics Committee of the Scientific Council of “Aghia Sophia” Children’s University Hospital (7516/01-04-19). Whole blood samples were collected from a total of 38 children, including 19 pediatric patients and 19 corresponding healthy controls, aged between 0.6 and 16 years, at the Haemophilia Center for Children and Adolescents/Haemostasis and Thrombosis Unit. Necessary criteria for participation were that the concerned neonates, children, and adolescents were hospitalized after presenting radiologically confirmed thrombotic episodes. Children affected by systemic comorbidities, associated with the development of thrombotic episodes, including malignancy and cardiovascular defects, were excluded from the study cohort. Both the patient and control groups comprised neonates, children, and adolescents, thereby representing the complete spectrum of pediatric thrombosis. The included patients were enrolled during follow-up visits subsequent to the thrombotic event. Within the control population, participants were clinically healthy and demonstrated a clear medical history of thrombosis. Following thorough discussion with the participants’ families, written informed consent was provided by the parents (or guardians) for the purposes of the study. The latter included the obtainment of a complete family history of four generations, demographic, stress-related and clinical information, as well as blood collection for molecular analyses. Blood samples were collected using clot activator-containing tubes, suitable for serum separation, and EDTA-coated tubes, intended for DNA extraction, respectively.

### 2.2. Perceived Stress Scale

The Perceived Stress Scale (PSS-14), validated in Greek [35], was used to assess the level of perceived stress. The PSS-14 is a 14-item self-report instrument that measures the degree in which individuals appraise situations in their lives as stressful. Participants rate the frequency of their feelings and thoughts during the previous month using a 5-point Likert Scale ranging from 0 (never) to 4 (very often). The scale comprises of seven positively worded and seven negatively worded items. Total scores are calculated by summing the scores of all items after reverse-coding the positively worded items, yielding a possible range from 0 to 56. Higher scores indicate greater levels of perceived stress experienced during the preceding month. The Greek version of the PSS-14 has demonstrated satisfactory psychometric properties in the Greek population [36].

### 2.3. Bioinformatic Identification of Candidate Stress-Specific miRNAs

An integrative bioinformatic approach was employed to identify miRNAs specific to childhood stress, focusing on the context of early-life stress attributed to adverse childhood experiences (ACEs) and early-life adversity (ELA). This framework incorporating ACEs and ELA was selected, since adversity in childhood represents a well-defined, objectively documented and biologically relevant model of early-life stress, thereby minimizing subjectivity associated with perceived stress measures and providing a reliable basis for the identification and prioritization of molecular candidates with genuine stress-specific relevance.

Initially, a systematic literature search was conducted in PubMed database, in order to retrieve experimental studies reporting significantly altered miRNA expression in early-life abuse, neglect, or other forms of stress and adversity. The applied keyword combinations included “microRNAs AND adverse childhood experience”, “microRNAs AND ACEs”, “microRNAs AND childhood adversity”, “microRNAs AND child abuse”, and “microRNAs AND early life adversity”, resulting in 26, 5, 12, 16 and 18 PubMed records, respectively. Studies were included according to the following criteria: (a) experimental evaluation of miRNA expression levels in biological specimens, including saliva, blood, sperm, tissue, urine, and cerebrospinal fluid (CSF); (b) reporting of distinct miRNA expression patterns, with a statistically significant difference between subjects exposed to stressful experiences/ACEs and controls with no reported exposure to equivalent stress. Studies were excluded from the analysis when they lacked a clearly defined miRNA expression pattern or did not demonstrate statistically significant differences in miRNA expression. The above process, following deduplication, resulted in 24 unique studies, screened by two researchers, and the construction of a dataset of miRNAs that are shown to exhibit altered expression upon ACEs and stress exposure related to ELA.

Thereafter, two customized gene panels specific to childhood stress was developed in a stepwise approach and served as the genomic dataset for subsequent analyses. The initial panel included 75 genes with an established association to childhood adversity, based on the relevant studies by Levine et al. and Damaskopoulou et al. [37,38]. From those genes, those experimentally shown to be regulated by at least one of the previous set of identified miRNAs were selected to comprise the gene panel for the final step of the analysis. The experimentally validated interactions were retrieved using TarBase v9.0 (DIANA Lab, University of Thessaly, Volos, Greece), as included in Supplementary Table S1. The yielded 31 genes included *COMT*, *NFKB1*, *FKBP5*, *HTR2A*, *BDNF*, *CRHR1*, *CRHR2*, *NR3C1*, *MAOA*, *GABRA2*, *NR3C2*, *DRD4*, *OXTR*, *JUN*, *RELA*, *FOSL2*, *JUNB*, *IRF2*, *JUND*, *PTGS2*, *MX1*, *OAS2*, *IFI16*, *FOSB*, *IRF7*, *MX2*, *PTGS1*, *FOS*, *IFIT1*, *IL1A* and *IFI27*, which are associated with childhood adversity, stress responsivity, immune regulation, inflammation, as well as neurobiological adaptation.

Finally, an additional level of biological specificity was introduced to reveal the most stress-specific candidate miRNAs among those retrieved in the initial step, which have already been shown to be associated with ACEs and ELA in the literature. This was performed through the identification of miRNAs simultaneously regulating the expression of multiple genes (more than one) in the final customized 31-gene panel, which was used as genomic input. Only experimentally validated interactions from direct experiments, such as luciferase reporter assays and cross linking and immunoprecipitation (CLIP)-based approaches, were retained for further analysis, but not indiscriminately across all cell types. In order to enhance biological relevance, the analysis solely included interactions demonstrated in human brain tissue, and particularly cerebral regions that are implicated in stress regulation, emotional processing, memory, and trauma-related neurobiology. Finally, validated gene targets were retrieved for each miRNA, and a corresponding target score was calculated based on the number of adversity-related genes it regulates in the selected brain tissue specimens. All eligible experimentally validated interactions meeting the predefined combinatory filtering criteria were treated equally in the analysis, without the application of additional weighting.

This approach enabled the prioritization of miRNAs with potential regulatory roles in biological pathways associated with childhood adversity and supported the construction of a biologically relevant stress-specific candidate miRNA dataset tailored to childhood. Among the identified miRNAs, those regulating the expression of over 65% of the genes comprising the final stress-specific panel, while also having been shown to regulate the expression of *IL1B* gene, which is considered a potential molecular bridge between stress-induced inflammation and thrombotic processes, were selected for experimental study in serum samples of PT patients and healthy controls, thereby allowing us to reliably investigate the potential link between these two conditions.

### 2.4. Serum RNA Extraction and Reverse Transcription

After 15–30 min of clotting at room temperature, the samples with clot activator were centrifuged for 10 min at 3000 rpm at 4 °C. Serum samples were visually inspected for evidence of hemolysis prior to the analysis, and none showed hemolysis-indicative discoloration. The supernatant serum phase was carefully collected and centrifuged again to ensure sample purity. Subsequently, total RNA extraction was performed from 300 μL of serum, using the NucleoSpin miRNA Plasma Mini kit (Macherey-Nagel GmbH, Düren, Germany), according to the manufacturer’s instructions for serum samples. During the lysis step of the RNA-extraction protocol, synthetic cel-miR-39-3p miRNA (miRBase accession number: MIMAT0000010) was included as a spike-in control to monitor the consistency of RNA extraction and downstream analyses (Qiagen, Hilden, Germany).

A BioSpec-nano Spectrophotometer for Life Science (Shimadzu Corporation, Kyoto, Japan) was used to assess concentration (ng/μL) and purity of the RNA samples, which had directly undergone subsequent reverse-transcribed into cDNA, following concentration normalization for maximum comparability. Polyadenylation and miRNA reverse transcription (RT) was performed in a one-step reaction using the miRCURY LNA RT Kit (Qiagen, Hilden, Germany). The 10 μL reaction involved the addition of 2 μL 5× RT-SYBR-Green Reaction Buffer, 2 μL RNA template, 1 μL 10× RT-Enzyme-Mix and 5 μL of nuclease-free water and was performed on a Gradient Thermal Cycler (Takara Bio, Shiga, Japan). Thermocycling incorporated a 60 min incubation step at 40 °C, subsequent inactivation at 95 °C and a final hold at 4 °C. The resulting cDNA samples were stored at −20 °C overnight until thawing and processing on the following day.

### 2.5. miRNA Profiling

Quantification of miR-34a-5p was carried out by real-time quantitative PCR (RT-qPCR), based on SYBR^®^ Green chemistry and performed on a LightCycler 480II instrument (Roche, Basel, Switzerland). Each 10 μL reaction contained 5 μL of 2× miRCURY SYBR-Green Master Mix, 4 μL cDNA, and 1 μL miRCURY LNA miRNA PCR Assay (Qiagen, Hilden, Germany). The cycling protocol involved a step of heat activation at 95 °C for 10 min, followed by 45 cycles of 95 °C for 15 s and 60 °C for 1 min, as well as a melting curve analysis step from 60 °C to 95 °C (0.03 °C/s). Following the standardization of the methodology and the use of a well-optimized assay, qRT-PCR reactions were performed in technical duplicate and measurements were considered acceptable when the Ct values did not vary by more than 0.5 cycles between replicates, while for the exogenous spike-in control, technical performance was considered acceptable when the Ct values did not vary by more than 0.5 cycles across the samples. The *Ct* values were obtained from the instrument’s built-in software by employing the “Absolute Quantification/2nd Derivative Max” analysis. Although the experimental protocol included 45 cycles, all samples reached amplification below 40 cycles, a cutoff threshold that was set to ensure reliable detection. The absolute quantification of the expression of miR-34a-5p, in copy number per μL of PCR reaction (copies/μL), required the construction of standard curves from custom design synthetic RNA oligonucleotides (Eurofins Genomics, Ebersberg, Germany) corresponding to the mature sequence of the miRNA (miRBase accession number: MIMAT0000255). The curve was produced by 2-fold serial dilutions of the reverse-transcribed oligonucleotides and demonstrated high linearity, with an R^2^ value of 0.9915 and PCR efficacy of 96%.

### 2.6. Genotyping for the IL1B +3953C/T (rs1143634) Polymorphism

Genomic DNA was isolated from the participants’ white blood cells using the NucleoSpin Blood kit (MACHEREY-NAGEL, Düren, Germany), according to the manufacturer’s instructions. The resulting DNA samples were subsequently genotyped for the functional single-nucleotide +3953C/T polymorphism of the *IL1B* gene (rs1143634). Genotyping was carried out by PCR amplification, performed using a Gradient Thermal Cycler (Takara Bio, Shiga, Japan), followed by digestion with the restriction endonuclease TaqI. The genotypes were determined by visualization under UV light, following the agarose gel electrophoresis of the resultant DNA fragments, facilitated by the use of GelRed fluorescent nucleic acid dye (Biotium, Fremont, CA, USA). Genotyping for the *IL1B* +3953C/T polymorphism and thermocycling included an initial denaturation at 94 °C for 5 min, followed by 35 cycles of denaturation at 94 °C for 50 s, annealing at 55 °C for 30 s, elongation at 72 °C for 30 s, and a final elongation step at 72 °C for 5 min. The PCR amplification resulted in a DNA fragment of 250 bp. The primers used for the target sequence were as follows: F: 5′-GTTGTCATCAGACTTTGACC-3′ and R: 5′-TTCAGTTCATATGGACCAGA-3′ [39]. The yielded PCR products were subsequently subjected to overnight incubation with the TaqI restriction enzyme at 65 °C, resulting in two fragments of 136 bp and 114 bp, in the presence of the prevalent C allele. After enzyme incubation, an intact DNA fragment of 250 bp indicated the presence of the minor T allele, which is associated with increased gene expression and raised circulating levels of proinflammatory and stress-related IL-1β.

### 2.7. Statistical Analysis

The statistical analyses were performed using SPSS Statistics (IBM Corp., Armonk, NY, USA), version 31.0.2.0. Normality was evaluated using the Shapiro–Wilk test for continuous values, based on the sample size of each cohort and respective subgroups. Comparisons between independent groups were conducted by employing the independent-samples *t*-test or the Mann–Whitney U test, in accordance with the distribution of values. Comparisons between more than two groups were performed using one-way ANOVA or the Kruskal–Wallis test. General linear models were used for the evaluation of the combined effects of different variables on miRNA levels. Comparative results are presented in bar charts, including error bars indicative of the variability around the estimated means. Categorical variables were compared between groups using the Pearson chi-square test. The control population was assessed for compliance with Hardy–Weinberg equilibrium, applying a chi-square test, for the genotypic frequencies of the *IL1B* +3953C/T polymorphism (*df* = 1). An a priori power analysis was performed to estimate the statistical power of the present study design (19 patients and 19 controls), assuming a 2-sided significance level of α = 0.05. According to the analysis, the study demonstrated 85.1% power to detect very large effects (Cohen’s d = 1). The level of statistical significance was set at *p* < 0.05 (2-tailed). A Bonferroni correction was not applied, given the exploratory nature of the study and the limited number of predefined, biologically driven hypotheses and the potential for statistical power reduction and increased risk for type II error (false negatives) in the present relatively small-sized cohort.

## 3. Results

### 3.1. Demographic, Clinical and Genotypic Profiling of the Studied Cohorts

A total of 38 participants were enrolled in the present study, including 19 PT patients, and 19 healthy controls of corresponding age and gender. A summary of the demographic characteristics of all participants is shown in Table 1, in addition to their genotype for the +3953C/T functional polymorphism (rs1143634) of the *IL1B* gene. For the patient group, the site of thrombosis was additionally documented, and its distribution closely mirrored the frequencies reported in the relevant literature, thereby adequately representing the anatomical spectrum of pediatric thrombosis. Age was compared between patients and controls through the performance of a Mann–Whitney U test, which did not reveal a statistically significant difference (*p* = 0.124). Regarding the variable of gender, a Pearson chi-square test was performed, also not yielding significant differences (*p* = 0.163). Finally, as to the participant’s genotypes as to the +3953C/T polymorphism of the *IL1B* gene, the corresponding frequencies were nearly identical between patients and controls, with the majority of participants carrying the CC wild-type genotype, followed by the heterozygous CT genotype, while TT homozygosity was observed in only a small proportion of the sample (7.8%).

### 3.2. Bioinformatic Identification of Candidate Stress-Specific miRNAs

**Figure 1:**
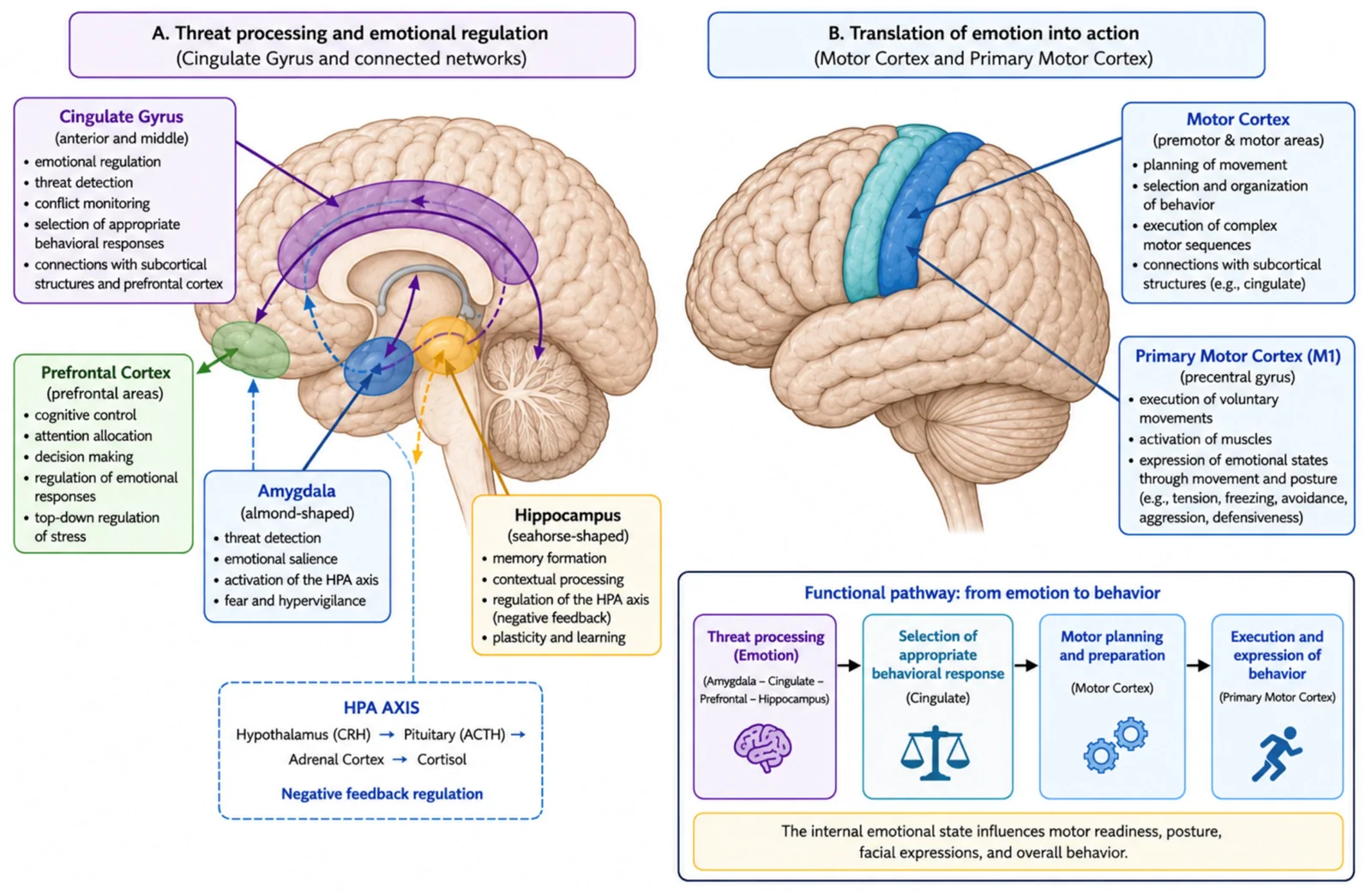
Schematic illustration of the brain regions, in which the miRNA/target interactions of interest were identified, as well as their relevance to emotional processing and stress-related responses. Upward arrows indicate upregulation, while downward arrows denote the downregulation of the respective miRNAs.

The systematic literature initially identified seven miRNAs whose dysregulation biological specimens, whether increased or decreased, have been associated with early-life stress and exposure to adverse childhood experiences. The final stress-specific candidate miRNA dataset included miRNAs with experimentally validated interactions with more than one of the genes comprising the 31-gene stress-specific panel, which have been demonstrated in human brain tissue, primarily from the entorhinal cortex, the primary motor cortex, and the motor cortex, all implicated in stress regulation, emotional processing, memory, and trauma-related neurobiology (Figure 1, Table 2).

Among the analyzed miRNAs, only miR-34a-5p was identified as the most promising stress-specific candidate in childhood, exhibiting the highest target coverage, with regulatory relationships with 80.6% of the genes comprising the final gene panel in the relevant stress- and emotion-related brain regions, while also having been shown to regulate the expression of the *IL1B* gene in epithelial cells, in a direct Photoactivatable-Ribonucleoside-Enhanced Crosslinking and Immunoprecipitation (PAR-CLIP) experiment (Supplementary Table S1). Hence, miR-34a-5p was bioinformatically selected for the experimental investigation of its candidate bridging stress-associated role between the contexts of inflammation and thrombosis.

### 3.3. Perceived Stress Scale (PSS-14)

The Perceived Stress Scale for Children (PSS-C, PSS-14 equivalent for children) questionnaire was collected in 17 of the cases of pediatric thrombosis and all 19 healthy controls by two trained members of the group, as shown in Table 3. The PSS-C is a simpler PSS-14 equivalent for children aged from 5 to 18 years [24,35,40]. For children aged between 0.6 and 4 years who were unable to independently complete the questionnaire, the mother answered the PSS-14; this was the case for 3 patients. Given that, in nine cases when both the children (aged 5–14) and their parents (most commonly mothers) provided responses (in PSS-C and PSS-14, respectively), there was no statistically significant difference in stress levels between them, it was considered acceptable to use the mothers’ responses as proxy for the 3 younger children below 5 years of age. The scales of perceived stress were considered to be low (score 1–15), moderate (score 16–30) and high (over 30), in accordance with previous studies [36].

Among the respondent children in both groups, most exhibited moderate perceived stress (8/17 or 47.1% of patients and 10/19 or 52.6% of controls), while very few of them had high perceived stress (2/17 or 11.8% of patients and 5/19 or 26.3% of controls). Overall, the PSS-14 results did not reveal any major difference in the levels of perceived stress between the groups of thrombotic patients and healthy controls (Pearson chi-square test, *p* = 0.329).

### 3.4. Expression Profiling of Serum miR-34a-5p in Relation to Stress and IL1B Genotype

#### 3.4.1. miR-34a-5p Expression in Relation to Stress Levels

**Figure 2:**
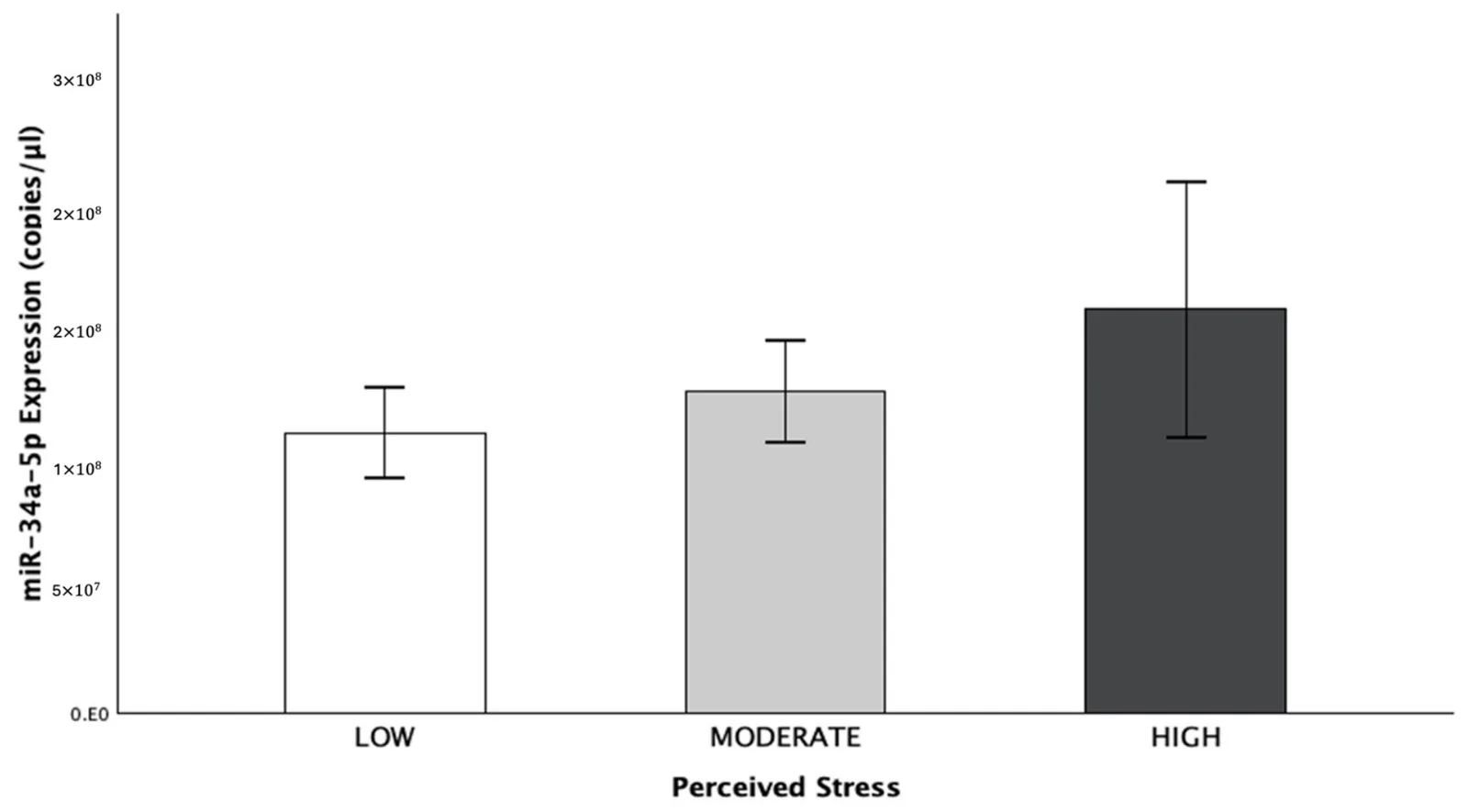
Mean expression (copies/μL) of miR-34a-5p across the three perceived stress categories within the overall study population, which did not significantly differ as to miRNA expression; however, a preliminary trend towards higher levels of miR-34a-5p with increasing stress was observed. Statistical significance was set at *p* < 0.05.

The expression of serum miR-34a-5p was evaluated in relation to stress levels initially in the overall study population and subsequently within each separate study group. Within the overall study cohort, serum miR-34a-5p levels did not significantly differ among the three categories according to the Kruskal–Wallis test (*p* = 0.961); however, a preliminary, clear visually observed trend towards increased expression was observed with increasing stress levels (Figure 2). The corresponding differences across the three stress categories within the independent groups of patients (*p* = 0.837) and controls (*p* = 0.727) also did not reach statistical significance according to the performed one-way ANOVA tests.

#### 3.4.2. miR-34a-5p Expression in Relation to IL1B Genotype

The genotype distribution in the control group (52.6% for CC, 36.8% for CT and 10.5% for TT; Table 1) was consistent with Hardy–Weinberg equilibrium (chi-square = 0.209; *p* = 0.650); thereby, the studied cohort was genuinely representative of the general population. The initially performed Pearson chi-square test did not reveal any significant differences between the patient and control groups (*p* = 0.827), with respect to the distribution of the three genotypes of the +3953C/T functional polymorphism, in exon 5 of the *IL1B* gene, which increases the levels of the circulating IL-1β in presence of the T allele. As the TT genotype group comprised only 3 children out of the total of 38 participants, the above *p* value concerning all three genotypes was used as an indication, and T homozygosity was excluded from the subsequent analyses to preserve statistical integrity.

**Figure 3:**
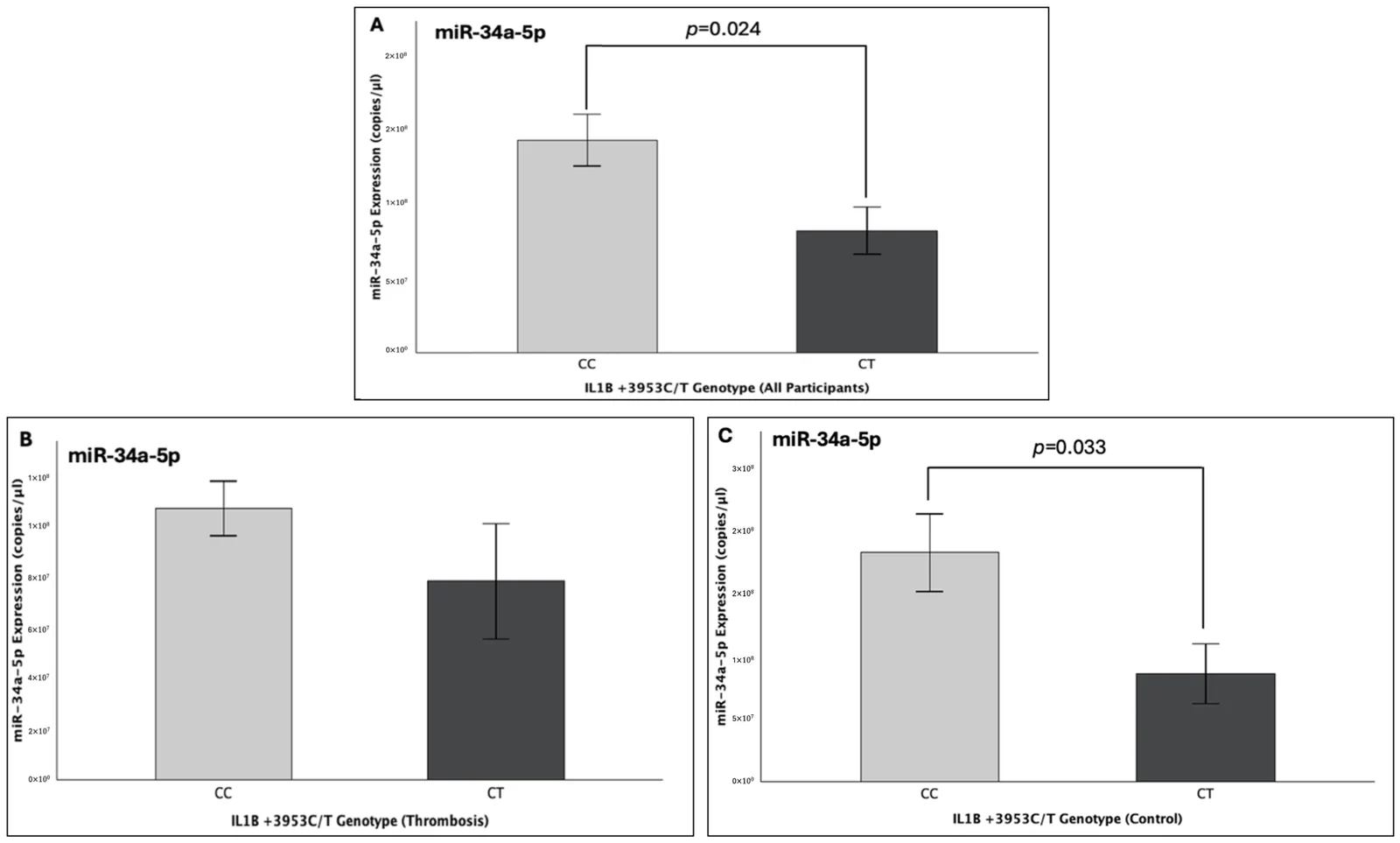
Comparative mean expression (copies/μL) of miR-34a-5p, which exhibited significant downregulation in presence of the T allele compared to the wild-type CC genotype of the *IL1B* 3953C/T functional polymorphism, in (**A**) the overall study population, (**B**) within the patient group and (**C**) within the control group, while demonstrating the same, yet not significant, trend in the group of patients with pediatric thrombosis. Statistical significance was set at *p* < 0.05.

Given the lack of statistically significant genotypic differences between the two groups, the expression of serum miR-34a-5p was subsequently evaluated in relation to the CC and CT genotypes of the *IL1B* +3953C/T in all participants and demonstrated a significant 43% reduction (FC:0.57; *p* = 0.024) in the presence of the T allele (Figure 3). The levels of miR-34a-5p were subsequently assessed in each separate group (patients and controls). In the group of PT, a non-significant, yet clear, trend for 27% reduced miR-34a-5p levels was observed in presence of the T allele (FC:0.73; *p* = 0.220), when compared to the CC wild-type genotype. However, within the control group, representing the general pediatric population, the same trend reached statistical significance, with a bold 53% reduction in the genotypic presence of the T allele (FC:0.47; *p* = 0.033) (Figure 3).

**Figure 4:**
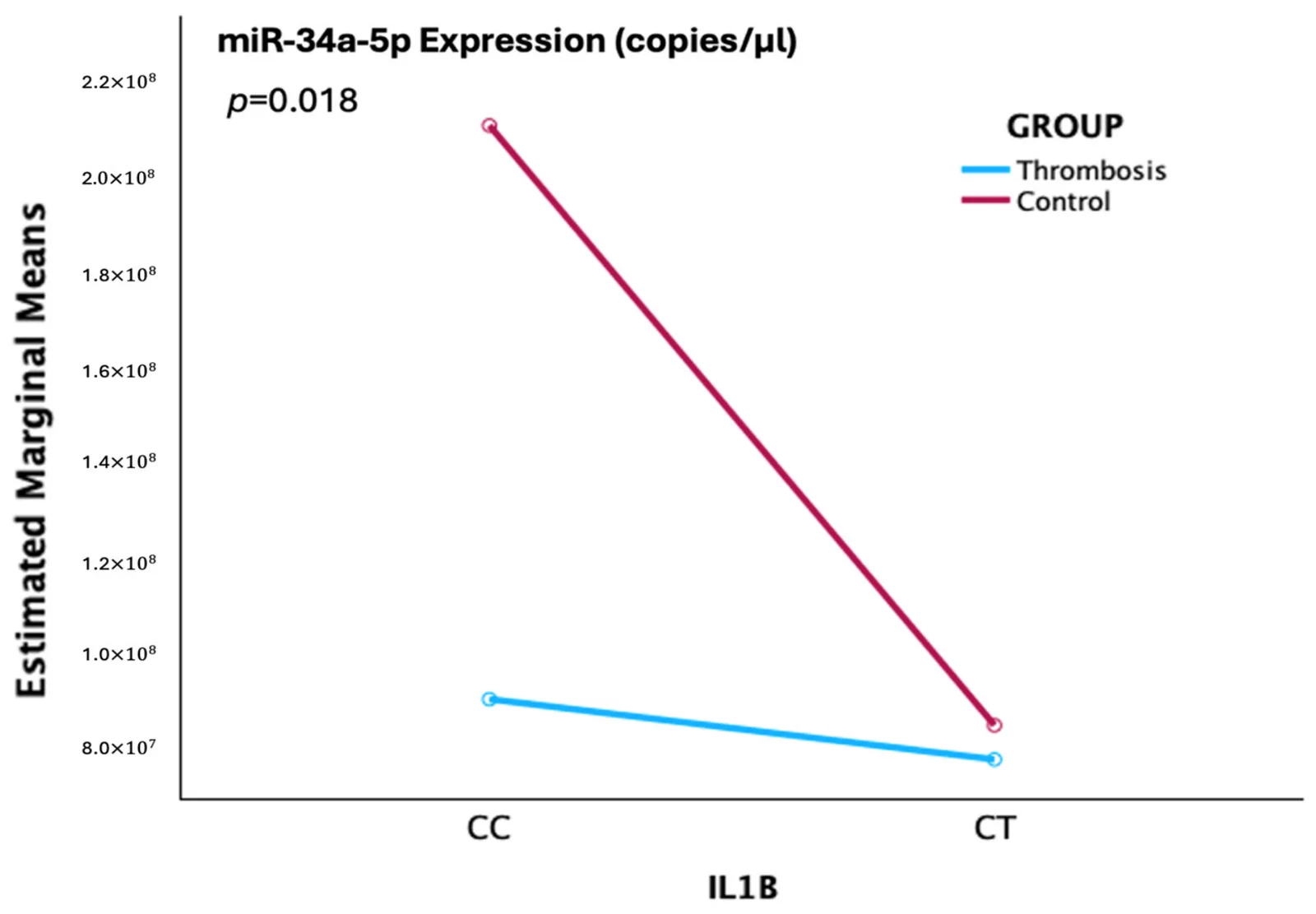
GLM analysis of miR-34a-5p expression (copies/μL), revealing a significant group-by-genotype interaction for the *IL1B* 3953C/T functional polymorphism, which results in the lowest miRNA levels in both groups, in presence of the T allele, which is associated with increased circulating IL-1β levels. Statistical significance was set at *p* < 0.05.

The latter finding was further supported by an additionally performed exploratory GLM analysis, which revealed a significant interaction between the groups (patients or controls) and the *IL1B* genotype as to the +3953C/T polymorphism on miR-34a-5p expression, with the lowest miRNA levels observed for the T allele carriers in both groups (*p* = 0.018) (Figure 4).

## 4. Discussion

Pediatric thrombosis represents a rare clinical entity with potentially life-threatening complications and a recently observed increasing incidence of up to 14 cases of blood clots in children and adolescents per 10,000 hospital admissions [1,2]. Multiple causative factors have been recognized, including immature balance of hemostasis, genetic variants conferring increased risk for thrombosis, use of central venous catheters, trauma, cancer, and, notably, inflammation [1]. Interestingly, accumulating evidence indicates an interaction between coagulation and inflammation in children and in adults, while all established risk factors share stress as the common denominator [1,7].

The link between stress and thrombosis appears to lie in stress-induced inflammation, which is triggered by acute or chronic stressors and contributes to the establishment of a prothrombotic state, thereby inducing hypofibrinolysis and hypercoagulability [9,10]. At a molecular level, this procoagulant inflammatory state is largely characterized by elevated levels of proinflammatory cytokines, particularly interleukins, which act as the central mediators [9,11,12,14]. Among the mainly expressed interleukins, IL-1β represents a promising candidate, since its systematic upregulation induces vascular permeability and immune cell recruitment, which constitute the main basis for thrombotic development [7,11,13,17], while also showing resistance to indirect, GR-mediated regulation under the stress conditions that lead to HPA disturbance [18,19].

Despite emerging evidence, the relationship between thrombosis and stress-related inflammation remains highly unexplored in adults and even more so in the rare context of PT, which exhibits fundamental differences as to its pathogenetic mechanisms and hemostatic physiology [7]. In particular, in both the adult and pediatric settings, there is a complete lack of studies investigating this interplay through the molecular intermediaries that can bridge stress, inflammation and thrombosis. Among the most promising candidates for such an approach are miRNAs, key epigenetic regulators of gene expression, which modulate critical biological processes, such as inflammation, but also exhibit characteristic and measurable expression patterns across different conditions, including both stress and thrombosis [7,32].

**Figure 5:**
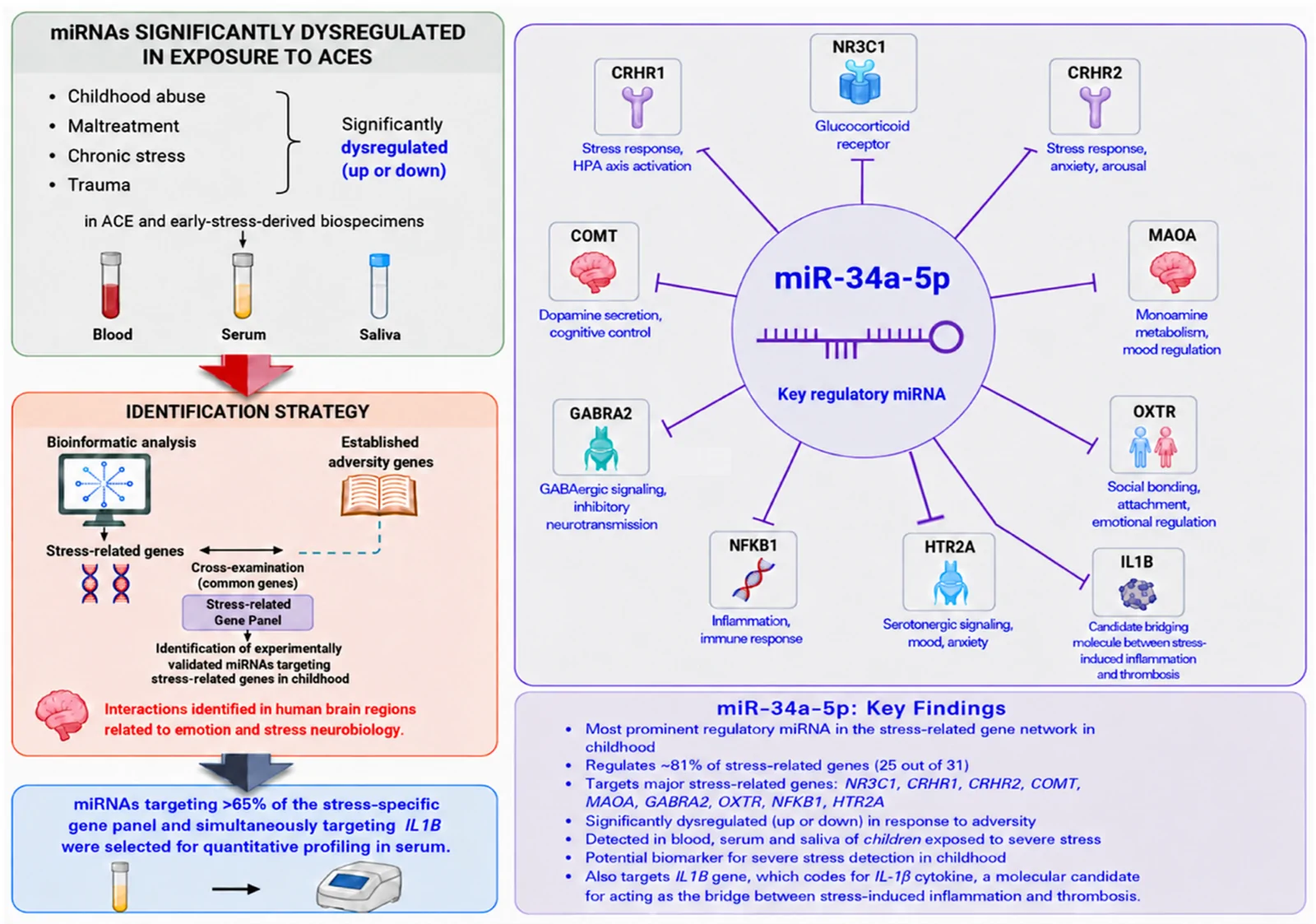
Summary and schematic overview of the principal stress-related gene targets of miR-34a-5p, the relevant biological functions of their encoded proteins, additionally including a concise flowchart illustrating the candidate stress-specific miRNA identification and experimental strategy.

Here, we aimed to explore the potential link between stress-induced inflammation and pediatric thrombosis through a combinatorial stepwise approach, integrating both bioinformatic and experimental work. Initially, we identified stress-specific candidate miRNAs in childhood, through a multifactorial bioinformatic approach, leveraging on the genetic basis of early life stress together with the available data on miRNA dysregulation patterns in children exposed to adverse childhood experiences (ACEs) and severe stress. The bioinformatic analysis yielded miR-34a-5p as the most specific dysregulated candidate miRNA for childhood stress, since it is experimentally shown to regulate the expression of 80.6% of the genes associated with childhood adversity, while showing dual and context-dependent dysregulation in ACE-related biological specimens. The regulatory relationships between miR-34a-5p and its target stress-associated genes have been demonstrated in brain tissue from regions involved in emotional regulation and stress-related responses, such as the entorhinal cortex, the primary motor cortex, and the motor cortex (Figure 5).

The expression of miR-34a-5p was subsequently evaluated in serum samples of 19 PT patients and 19 healthy controls in both a comparative and integrative manner. In fact, the levels of miR-34a-5p were evaluated within each group, but also in the overall study population, in relation to perceived stress levels, measured by the PSS-14 questionnaire. MiRNA expression was also assessed in relation to the genotypes of the participants as to the +3953C/T functional genetic polymorphism, in exon 5 of the *IL1B* gene, which, in the presence of the T allele, is associated with increased circulating levels of the proinflammatory and stress-related IL-1β [41]. The *IL1B* +3953C/T has also been shown to influence stress-related inflammatory conditions, such as rheumatoid arthritis and systemic sclerosis [42,43].

While no significant difference was observed either in stress levels or the genotypes for *IL1B* +3953C/T alone between PT patients and controls, miRNA levels in relation to these factors revealed interesting findings. With respect to perceived stress, miR-34a-5p exhibited a clear trend towards upregulation in light of increasing stress levels, which, however, did not meet statistical significance but served as a hypothesis-generating pattern. More specifically, this trend possibly reflects a moderate, possibly reactive increase in miR-34a-5p expression, in order to regulate its overexpressed targets under stressful conditions.

As to miR-34a-5p expression in relation to *IL1B* genotype for the +3953C/T polymorphism, analysis of the overall study population revealed a significant 43% decrease in miRNA levels, in presence of the T allele (FC:0.57; *p* = 0.024), which is known to increase proinflammatory IL1β levels in the bloodstream [41]. The same trend was observed within the individual study groups of patients and controls. Interestingly, patients showed a non-significant 27% reduction in miR-34a-5p expression in the presence of the T allele (FC:0.73; *p* = 0.220), whereas, in the control population, this reduction was statistically significant and particularly pronounced, reaching 53% (FC:0.47; *p* = 0.033). These preliminary findings were further supported as a significant interaction was identified between the health status of the participants (patients or controls) and *IL1B* for the +3953C/T polymorphism, with both groups exhibiting the lowest point of miR-34a-5p expression in presence of the T allele (*p* = 0.018).

The above exploratory findings highlight the distinct nature of pediatric thrombosis compared to the adult form, as psychological stress did not appear to play a significant role in its pathogenesis, thereby suggesting that thrombotic susceptibility in childhood might be predominantly driven by biological risk factors rather than perceived stress itself. However, the observed non-significant yet clear trend toward higher expression of the candidate stress-associated miR-34a-5p across increasing stress suggests that stress might still exert some measurable biological effects. Moreover, given the questionnaire-based nature of stress assessment and PSS-14 limitations, particularly in younger children with doubtful self-reporting reliability, this finding should be cautiously interpreted as perceived stress differs from biologically translatable stress, and it might indicate that the observed trend would be more pronounced if biological markers, such as cortisol, were measured.

Finally, the significant downregulation of miR-34a-5p expression in presence of the T allele of the *IL1B* +3953C/T polymorphism, which increases the circulating proinflammatory interleukin, also reflects the stronger influence of biological determinants within pediatric populations. This particular functional polymorphism served as a genetically induced proxy for a biological context of increased IL-1β levels, in presence of the T allele, thereby providing an additional layer of evidence beyond subjective stress assessment with the PSS-14 questionnaire. In this context, the dynamic interplay between miRNA levels and genotype may help shed light on how miR-34a-5p responds under conditions of enhanced interleukin-mediated inflammation, such as severe stress, which is characterized by a significant and long-lasting increase in IL-1β.

Considering that miR-34a-5p was yielded as a stress-specific candidate in childhood and is experimentally validated to regulate the expression of the *IL1B* gene, the observed significant decrease in its expression under the condition of genetically predefined *IL1B* upregulation may suggest a potential regulatory mechanism in which this miRNA is led to functional exhaustion in light of an overexpressing target. Hence, miR-34a-5p appears to be a promising candidate for bridging the biological processes of stress-related inflammation and thrombosis and its decrease might possibly be indicative of underlying prothrombotic IL-1β upregulation. The present pilot study provides a preliminary indication of the potential value of miRNAs as molecular markers for the investigation of the links between conditions such as stress, thrombosis and inflammation.

Moreover, the present hypothesis-generating findings can be supportive of further investigations into miR-34a-5p in larger pediatric populations in the context of stress-induced inflammation in PT. Since the upregulation of IL-1β levels leads to an inflammatory and prothrombotic environment, the downregulation of serum miR-34a-5p might be indicative of a procoagulant environment with ongoing stress-induced inflammation. This notion is additionally supported by the established short- and long-term downregulation of miR-34a-5p in the serum of patients with PT compared to healthy children [7]. It is also suggested that the role of miR-34a-5p in pediatric thrombosis may extend beyond disease status itself, and that might be part of a multifactorial prothrombotic state, also affected by stress-induced inflammation.

Finally, given the results of the present bioinformatic protocol that yielded miR-34a-5p as a stress-specific candidate in childhood due to its reported dysregulation and key stress-related target genes, as well as its indicated dynamic interplay with IL-1β levels, it could also serve as a potential stress-associated miRNA. Further studies could evaluate its potential in bridging stress-induced inflammation and thrombosis, following the proper validation of its direct interplay with IL-1β levels and possibly other molecular inflammatory indicators, or other PT-related variables, in larger independent populations. In addition, its candidate stress-specific role, revealed in the bioinformatic part of this study, may be particularly valuable in the context of ACEs and severe early-life stress, where stress might remain undetected due to the limitations of interview- or questionnaire-based evaluation approaches.

### Study Limitations

The statistical power of the present study is limited for non-large effects due to the relatively small cohort and subgroup sizes, which may be attributed to the rare nature of PT. Therefore, larger cohorts have to be studied before definite biological conclusions can be drawn, especially in the case of observed preliminary trends, such as the upregulation of miR-34a-5p in light of increasing stress or its decreasing expression in presence of the T allele within the PT group. A larger study cohort will allow for better representation of the TT genotype and surpass the limitations of the present study, which was restricted to a CC-versus-CT comparison due to the very low frequency of TT carriers. Hence, the observed significant preliminary association should be interpreted with caution since it does not establish a definite or generalized T-allele effect.

Another limitation lies in the approach for stress evaluation, which was based on the PSS-14 questionnaire of perceived stress during a specific time period and therefore might underestimate the degree of stress exposure, which can exert biological effects. Hence, the absence of a significant association between miR-34a-5p levels and subjectively perceived stress should be interpreted with caution. Finally, a further limitation lies in the fact that circulating IL-1β levels were not directly measured in the serum samples of the enrolled children. Therefore, although the +3953C/T functional polymorphism of the *IL1B* gene provides a proxy for genetically induced upregulation in IL-1β concentrations, this may only provide indirect biological support of a potential interplay between *IL1B* and miR-34a-5p expression rather than confirming an association between miRNA levels and corresponding changes in IL-1β concentrations, inflammatory activity or a subsequently induced prothrombotic state.

## 5. Conclusions

The present study initially revealed miR-34a-5p as a stress-specific candidate through a multistep bioinformatic filtering model, focused on the objective context of childhood adversity and stress-related neurobiological experimental data. Its experimental investigation into the serum of patients with pediatric thrombosis and healthy controls revealed no association with subjectively perceived stress; however, its expression was significantly downregulated in the presence of the T allele of the functional +3953C/T polymorphism of its target gene *IL1B*, associated with increased levels of the stress-induced proinflammatory and simultaneously prothrombotic IL-1β cytokine. Given the previously established long-term downregulation of miR-34a-5p in pediatric thrombosis, the preliminary findings of the present study indirectly support its dynamic relationship with *IL1B* and render miR-34a-5p a promising candidate molecule for bridging the biological processes of stress-associated inflammation and thrombosis.

## Figures and Tables

**Table 1:** Overview of the characteristics and thrombosis-related clinical data of the studied patient and control groups. Age and gender were statistically compared between the two groups. The level of statistical significance was set at *p* < 0.05. The two cohorts did not differ significantly as to age and gender. ^1^ Mean (range); ^a^ Mann–Whitney U test (2-sided), ^b^ Pearson chi-square (2-sided).

Variable	Patients (*n* = 19)		Controls (*n* = 19)		*p*-Value
**Age (Years) ^1^**	**9.51 (0.6–16)**		7.03 (0.6–15)		0.124 ^a^
**Gender** *n* (%)	Male	Female	Male	Female	0.163 ^b^
	15 (78.9%)	4 (21.1%)	11 (57.9%)	8 (42.1%)	
**Anatomical site of thrombosis**					
Cerebral *n* (%)		Lower limbs *n* (%)		Pulmonary *n* (%)	
9 (47.4%)		6 (31.6%)		4 (21.0%)	
***IL1B* +3953C/T (rs1143634) Genotype (*n*, %)**					
**Patients** *n* (%)	CC 11 (57.9%)	CT 7 (36.8%)	TT 1 (5.3%)		0.827 ^b^
**Controls** *n* (%)	CC 10 (52.6%)	CT 7 (36.8%)	TT 2 (10.5%)		

**Table 2:** Overview of the final results of the combinatorial bioinformatic analysis, employed for the identification of stress-specific candidate miRNAs in childhood. From the miRNAs that have been reported as dysregulated in ACE-related and ELA-related biospecimens, only miR-34a-5p was identified as the most stress-specific candidate, regulating the expression of approximately 81% of the genes included in the developed ACE-specific panel.

miRNA	Target Genes	Target Score
miR-34a-5p	*COMT*, *NFKB1*, *HTR2A*, *CRHR1*, *CRHR2*, *NR3C1*, *MAOA*, *GABRA2*, *NR3C2*, *OXTR*, *JUN*, *RELA*, *FOSL2*, *JUNB*, *IRF2*, *JUND*, *PTGS2*, *MX1*, *OAS2*, *IFI16*, *FOSB*, *IRF7*, *MX2*, *PTGS1*, *IFI27*	25/31 (80.6%)
miR-124-3p	*COMT*, *HTR2A*, *BDNF*, *NR3C1*, *GABRA2*, *NR3C2*, *RELA*, *FOSL2*, *IFIT1*	9/31 (29.0%)
miR-16-5p	*COMT*, *FKBP5*, *HTR2A*, *NR3C1*, *MAOA*, *GABRA2*, *NR3C2*, *DRD4*, *IFI16*	9/31 (29.0%)
miR-30a-5p	*NFKB1*, *NR3C1*, *MAOA*, *GABRA2*, *JUNB*, *JUND*, *PTGS2*, *MX1*, *OAS2*, *FOS*, *IFIT1*, *IL1A*	12/31 (38.7%)
miR-146a-5p	*HTR2A*, *GABRA2*, *FOSL2*, *JUND*	4/31 (12.9%)

**Table 3:** Overview of the distribution of stress-level categories in the two studied groups. ^a^ Pearson chi-square test (2-sided).

Group	Perceived Stress Levels (Based on the PSS-14 Questionnaire)			*p*-Value
Thrombosis	Low 7 (41.2%)	Moderate 8 (47.1%)	High 2 (11.8%)	0.329 ^a^
Control	Low 4 (21.1%)	Moderate 10 (52.6%)	High 5 (26.3%)	

## Data Availability

Original data presented in this research shall be available upon request to the corresponding author. Certain data may be subject to limited availability in accordance with confidentiality and privacy-protecting protocols or temporary ongoing study conduction.

## Supplementary Materials

The following supporting information can be downloaded at: https://www.mdpi.com/article/10.3390/genes17091124/s1, Supplementary Table S1: Key data of experimentally validated miRNA/target interactions yielded from the bioinformatic analysis.
