# Supplementary material for: Candidate Stress-Specific microRNA Expression in Pediatric Thrombosis and Healthy Children: A Comparative Analysis of Serum miR-34a-5p in Relation to Stress Levels and Inflammatory Genetic Traits
Source: Genes (Basel). 2026 Sep 16;17(9):1124. doi: 10.3390/genes17091124 (PMC13606060; doi:10.3390/genes17091124)
Supplement: Supplementary file 1 [file genes-17-01124-s001.zip › genes-4458963-supplementary.pdf]

**Supplementary Table S1:** Key data of experimentally validated miRNA/target interactions yielded from the bioinformatic analysis										
**Gene name**	**miRNA name**	**Tissue**	**Cell line**	**Experimental type**	**Phenotype**	**Binding site**	**Transcript name**	**Transcript id**	**Transcript location**	**Regulation**
COMT	hsa-miR-34a-5p	Brain - Cingulate gyrus	NA	Direct	Post-mortem	chr22 | + | 19962551-19962563	COMT-202	ENST00000361682	CDS	↓
COMT	hsa-miR-34a-5p	Brain - Cingulate gyrus	NA	Direct	Post-mortem	chr22 | + | 19968579-19968591	COMT-202	ENST00000361682	CDS	↓
COMT	hsa-miR-34a-5p	Brain - Motor cortex	NA	Direct	Post-mortem	chr22 | + | 19962551-19962563	COMT-202	ENST00000361682	CDS	↓
COMT	hsa-miR-34a-5p	Brain - Motor cortex	NA	Direct	Post-mortem	chr22 | + | 19962706-19962718	COMT-202	ENST00000361682	CDS	↓
COMT	hsa-miR-34a-5p	Brain - Motor cortex	NA	Direct	Post-mortem	chr22 | + | 19962765-19962777	COMT-202	ENST00000361682	CDS	↓
COMT	hsa-miR-124-3p	Brain - Cingulate gyrus	NA	Direct	Post-mortem	chr22 | + | 19962620-19962632	COMT-202	ENST00000361682	CDS	↓
COMT	hsa-miR-124-3p	Brain - Motor cortex	NA	Direct	Post-mortem	chr22 | + | 19962620-19962632	COMT-202	ENST00000361682	CDS	↓
COMT	hsa-miR-16-5p	Brain - Cingulate gyrus	NA	Direct	Post-mortem	chr22 | + | 19963603-19963615	COMT-202	ENST00000361682	CDS	↓
NFKB1	hsa-miR-30a-5p	Brain - Motor cortex	NA	Direct	Post-mortem	chr4 | + | 102578951-102578963	NFKB1-206	ENST00000507079	CDS	↓

Supplementary Table S1: Key data of experimentally validated miRNA/target interactions yielded from the bioinformatic analysis										
**Gene name**	**miRNA name**	**Tissue**	**Cell line**	**Experimental type**	**Phenotype**	**Binding site**	**Transcript name**	**Transcript id**	**Transcript location**	**Regulation**
NFKB1	hsa-miR-34a-5p	Brain - Cingulate gyrus	NA	Direct	Post-mortem	chr4 | + | 102529898-102529910	NFKB1-206	ENST00000507079	CDS	↓
NFKB1	hsa-miR-34a-5p	Brain - Cingulate gyrus	NA	Direct	Post-mortem	chr4 | + | 102613458-102613474	NFKB1-206	ENST00000507079	CDS	↓
NFKB1	hsa-miR-30a-5p	Brain - Motor cortex	NA	Direct	Post-mortem	chr4 | + | 102612076-102612088	NFKB1-206	ENST00000507079	CDS	↓
FKBP5	hsa-miR-16-5p	Brain - Cingulate gyrus	NA	Direct	Post-mortem	chr6 | - | 35642767-35642779	FKBP5-202	ENST00000536438	CDS	↓
HTR2A	hsa-miR-34a-5p	Brain - Motor cortex	NA	Direct	Post-mortem	chr13 | - | 46831842-46831854	HTR2A-202	ENST00000543956	3UTR	↓
HTR2A	hsa-miR-34a-5p	Brain - Motor cortex	NA	Direct	Post-mortem	chr13 | - | 46834217-46834229	HTR2A-202	ENST00000543956	3UTR	↓
HTR2A	hsa-miR-34a-5p	Brain - Motor cortex	NA	Direct	Post-mortem	chr13 | - | 46835468-46835480	HTR2A-202	ENST00000543956	CDS	↓
HTR2A	hsa-miR-146a-5p	Brain - Cingulate gyrus	NA	Direct	Post-mortem	chr13 | - | 46834788-46834800	HTR2A-202	ENST00000543956	3UTR	↓
HTR2A	hsa-miR-124-3p	Brain - Motor cortex	NA	Direct	Post-mortem	chr13 | - | 46834130-46834142	HTR2A-202	ENST00000543956	3UTR	↓

**Supplementary Table S1:** Key data of experimentally validated miRNA/target interactions yielded from the bioinformatic analysis										
**Gene name**	**miRNA name**	**Tissue**	**Cell line**	**Experimental type**	**Phenotype**	**Binding site**	**Transcript name**	**Transcript id**	**Transcript location**	**Regulation**
HTR2A	hsa-miR-124-3p	Brain - Cingulate gyrus	NA	Direct	Post-mortem	chr13 | - | 46832679-46832691	HTR2A-202	ENST00000543956	3UTR	↓
HTR2A	hsa-miR-124-3p	Brain - Cingulate gyrus	NA	Direct	Post-mortem	chr13 | - | 46832903-46832915	HTR2A-202	ENST00000543956	3UTR	↓
HTR2A	hsa-miR-124-3p	Brain - Cingulate gyrus	NA	Direct	Post-mortem	chr13 | - | 46834498-46834510	HTR2A-202	ENST00000543956	3UTR	↓
HTR2A	hsa-miR-146a-5p	Brain - Motor cortex	NA	Direct	Post-mortem	chr13 | - | 46832352-46832364	HTR2A-202	ENST00000543956	3UTR	↓
HTR2A	hsa-miR-146a-5p	Brain - Motor cortex	NA	Direct	Post-mortem	chr13 | - | 46834788-46834800	HTR2A-202	ENST00000543956	3UTR	↓
HTR2A	hsa-miR-16-5p	Brain - Cingulate gyrus	NA	Direct	Post-mortem	chr13 | - | 46832679-46832691	HTR2A-202	ENST00000543956	3UTR	↓
HTR2A	hsa-miR-16-5p	Brain - Cingulate gyrus	NA	Direct	Post-mortem	chr13 | - | 46832903-46832915	HTR2A-202	ENST00000543956	3UTR	↓
HTR2A	hsa-miR-16-5p	Brain - Cingulate gyrus	NA	Direct	Post-mortem	chr13 | - | 46834498-46834510	HTR2A-202	ENST00000543956	3UTR	↓
HTR2A	hsa-miR-146a-5p	Brain - Cingulate gyrus	NA	Direct	Post-mortem	chr13 | - | 46832352-46832364	HTR2A-202	ENST00000543956	3UTR	↓

**Supplementary Table S1:** Key data of experimentally validated miRNA/target interactions yielded from the bioinformatic analysis										
**Gene name**	**miRNA name**	**Tissue**	**Cell line**	**Experimental type**	**Phenotype**	**Binding site**	**Transcript name**	**Transcript id**	**Transcript location**	**Regulation**
BDNF	hsa-miR-124-3p	Brain - Motor cortex	NA	Direct	Post-mortem	chr11 | - | 27658260-27658272	BDNF-201	ENST00000314915	CDS	↓
CRHR1	hsa-miR-34a-5p	Brain - Motor cortex	NA	Direct	Post-mortem	chr17 | + | 45833735-45833747	CRHR1-213	ENST00000619154	3UTR	↓
CRHR2	hsa-miR-34a-5p	Brain - Motor cortex	NA	Direct	Post-mortem	chr7 | - | 30655682-30655694	CRHR2-201	ENST00000341843	CDS	↓
NR3C1	hsa-miR-34a-5p	Brain - Cingulate gyrus	NA	Direct	Post-mortem	chr5 | - | 143277974-143277986	NR3C1-202	ENST00000343796	3UTR	↓
NR3C1	hsa-miR-34a-5p	Brain - Cingulate gyrus	NA	Direct	Post-mortem	chr5 | - | 143279458-143279470	NR3C1-202	ENST00000343796	3UTR	↓
NR3C1	hsa-miR-34a-5p	Brain - Cingulate gyrus	NA	Direct	Post-mortem	chr5 | - | 143310144-143310156	NR3C1-202	ENST00000343796	CDS	↓
NR3C1	hsa-miR-34a-5p	Brain - Cingulate gyrus	NA	Direct	Post-mortem	chr5 | - | 143399885-143399897	NR3C1-202	ENST00000343796	CDS	↓
NR3C1	hsa-miR-34a-5p	Brain - Cingulate gyrus	NA	Direct	Post-mortem	chr5 | - | 143399932-143399944	NR3C1-202	ENST00000343796	CDS	↓
NR3C1	hsa-miR-34a-5p	Brain - Cingulate gyrus	NA	Direct	Post-mortem	chr5 | - | 143400018-143400030	NR3C1-202	ENST00000343796	CDS	↓

**Supplementary Table S1:** Key data of experimentally validated miRNA/target interactions yielded from the bioinformatic analysis										
**Gene name**	**miRNA name**	**Tissue**	**Cell line**	**Experimental type**	**Phenotype**	**Binding site**	**Transcript name**	**Transcript id**	**Transcript location**	**Regulation**
NR3C1	hsa-miR-34a-5p	Brain - Cingulate gyrus	NA	Direct	Post-mortem	chr5 | - | 143400389-143400401	NR3C1-202	ENST00000343796	CDS	↓
NR3C1	hsa-miR-34a-5p	Brain - Cingulate gyrus	NA	Direct	Post-mortem	chr5 | - | 143400389-143400401	NR3C1-202	ENST00000343796	CDS	↓
NR3C1	hsa-miR-34a-5p	Brain - Motor cortex	NA	Direct	Post-mortem	chr5 | - | 143281374-143281386	NR3C1-202	ENST00000343796	3UTR	↓
NR3C1	hsa-miR-34a-5p	Brain - Motor cortex	NA	Direct	Post-mortem	chr5 | - | 143281883-143281895	NR3C1-202	ENST00000343796	CDS	↓
NR3C1	hsa-miR-34a-5p	Brain - Motor cortex	NA	Direct	Post-mortem	chr5 | - | 143281883-143281895	NR3C1-202	ENST00000343796	3UTR	↓
NR3C1	hsa-miR-34a-5p	Brain - Motor cortex	NA	Direct	Post-mortem	chr5 | - | 143310144-143310156	NR3C1-202	ENST00000343796	CDS	↓
NR3C1	hsa-miR-34a-5p	Brain - Motor cortex	NA	Direct	Post-mortem	chr5 | - | 143400389-143400401	NR3C1-202	ENST00000343796	CDS	↓
NR3C1	hsa-miR-30a-5p	Brain - Cingulate gyrus	NA	Direct	Post-mortem	chr5 | - | 143281423-143281435	NR3C1-202	ENST00000343796	3UTR	↓
NR3C1	hsa-miR-30a-5p	Brain - Motor cortex	NA	Direct	Post-mortem	chr5 | - | 143279935-143279947	NR3C1-202	ENST00000343796	3UTR	↓

**Supplementary Table S1:** Key data of experimentally validated miRNA/target interactions yielded from the bioinformatic analysis										
**Gene name**	**miRNA name**	**Tissue**	**Cell line**	**Experimental type**	**Phenotype**	**Binding site**	**Transcript name**	**Transcript id**	**Transcript location**	**Regulation**
NR3C1	hsa-miR-124-3p	Brain - Cingulate gyrus	NA	Direct	Post-mortem	chr5 | - | 143281881-143281893	NR3C1-202	ENST00000343796	CDS	↓
NR3C1	hsa-miR-124-3p	Brain - Cingulate gyrus	NA	Direct	Post-mortem	chr5 | - | 143281881-143281893	NR3C1-202	ENST00000343796	3UTR	↓
NR3C1	hsa-miR-124-3p	Brain - Motor cortex	NA	Direct	Post-mortem	chr5 | - | 143280504-143280516	NR3C1-202	ENST00000343796	3UTR	↓
NR3C1	hsa-miR-124-3p	Brain - Motor cortex	NA	Direct	Post-mortem	chr5 | - | 143281881-143281893	NR3C1-202	ENST00000343796	CDS	↓
NR3C1	hsa-miR-124-3p	Brain - Motor cortex	NA	Direct	Post-mortem	chr5 | - | 143281881-143281893	NR3C1-202	ENST00000343796	3UTR	↓
NR3C1	hsa-miR-30a-5p	Brain - Motor cortex	NA	Direct	Post-mortem	chr5 | - | 143280145-143280157	NR3C1-202	ENST00000343796	3UTR	↓
NR3C1	hsa-miR-16-5p	Brain - Motor cortex	NA	Direct	Post-mortem	chr5 | - | 143279137-143279149	NR3C1-202	ENST00000343796	3UTR	↓
MAOA	hsa-miR-34a-5p	Brain - Cingulate gyrus	NA	Direct	Post-mortem	chrX | + | 43731316-43731328	MAOA-204	ENST00000542639	CDS	↓
MAOA	hsa-miR-34a-5p	Brain - Motor cortex	NA	Direct	Post-mortem	chrX | + | 43731365-43731377	MAOA-204	ENST00000542639	CDS	↓

**Supplementary Table S1:** Key data of experimentally validated miRNA/target interactions yielded from the bioinformatic analysis										
**Gene name**	**miRNA name**	**Tissue**	**Cell line**	**Experimental type**	**Phenotype**	**Binding site**	**Transcript name**	**Transcript id**	**Transcript location**	**Regulation**
MAOA	hsa-miR-34a-5p	Brain - Motor cortex	NA	Direct	Post-mortem	chrX | + | 43731724-43731736	MAOA-204	ENST00000542639	CDS	↓
MAOA	hsa-miR-34a-5p	Brain - Motor cortex	NA	Direct	Post-mortem	chrX | + | 43745521-43745533	MAOA-204	ENST00000542639	3UTR	↓
MAOA	hsa-miR-16-5p	Brain - Cingulate gyrus	NA	Direct	Post-mortem	chrX | + | 43741993-43742005	MAOA-204	ENST00000542639	CDS	↓
MAOA	hsa-miR-30a-5p	Brain - Motor cortex	NA	Direct	Post-mortem	chrX | + | 43740725-43740737	MAOA-204	ENST00000542639	CDS	↓
GABRA2	hsa-miR-34a-5p	Brain - Cingulate gyrus	NA	Direct	Post-mortem	chr4 | - | 46249025-46249037	GABRA2-209	ENST00000510861	3UTR	↓
GABRA2	hsa-miR-34a-5p	Brain - Cingulate gyrus	NA	Direct	Post-mortem	chr4 | - | 46250224-46250236	GABRA2-209	ENST00000510861	3UTR	↓
GABRA2	hsa-miR-34a-5p	Brain - Cingulate gyrus	NA	Direct	Post-mortem	chr4 | - | 46262048-46262060	GABRA2-209	ENST00000510861	CDS	↓
GABRA2	hsa-miR-34a-5p	Brain - Cingulate gyrus	NA	Direct	Post-mortem	chr4 | - | 46303520-46303532	GABRA2-209	ENST00000510861	CDS	↓
GABRA2	hsa-miR-34a-5p	Brain - Cingulate gyrus	NA	Direct	Post-mortem	chr4 | - | 46312546-46312558	GABRA2-209	ENST00000510861	CDS	↓

**Supplementary Table S1:** Key data of experimentally validated miRNA/target interactions yielded from the bioinformatic analysis										
**Gene name**	**miRNA name**	**Tissue**	**Cell line**	**Experimental type**	**Phenotype**	**Binding site**	**Transcript name**	**Transcript id**	**Transcript location**	**Regulation**
GABRA2	hsa-miR-34a-5p	Brain - Primary Visual Cortex	NA	Direct	Post-mortem	chr4 | - | 46312546-46312558	GABRA2-209	ENST00000510861	CDS	↓
GABRA2	hsa-miR-146a-5p	Brain - Cingulate gyrus	NA	Direct	Post-mortem	chr4 | - | 46249235-46249247	GABRA2-209	ENST00000510861	3UTR	↓
GABRA2	hsa-miR-146a-5p	Brain - Motor cortex	NA	Direct	Post-mortem	chr4 | - | 46249235-46249247	GABRA2-209	ENST00000510861	3UTR	↓
GABRA2	hsa-miR-124-3p	Brain - Cingulate gyrus	NA	Direct	Post-mortem	chr4 | - | 46303534-46303546	GABRA2-209	ENST00000510861	CDS	↓
GABRA2	hsa-miR-124-3p	Brain - Cingulate gyrus	NA	Direct	Post-mortem	chr4 | - | 46303534-46303546	GABRA2-209	ENST00000510861	CDS	↓
GABRA2	hsa-miR-30a-5p	Brain - Primary Motor Cortex	NA	Direct	Post-mortem	chr4 | - | 46249337-46249349	GABRA2-209	ENST00000510861	3UTR	↓
GABRA2	hsa-miR-16-5p	Brain - Motor cortex	NA	Direct	Post-mortem	chr4 | - | 46262027-46262039	GABRA2-209	ENST00000510861	CDS	↓
NR3C2	hsa-miR-124-3p	Brain - Motor cortex	NA	Direct	Post-mortem	chr4 | - | 148152537-148152549	NR3C2-202	ENST00000344721	CDS	↓

Supplementary Table S1: Key data of experimentally validated miRNA/target interactions yielded from the bioinformatic analysis

Gene name	miRNA name	Tissue	Cell line	Experimental type	Phenotype	Binding site	Transcript name	Transcript id	Transcript location	Regulation
NR3C2	hsa-miR-124-3p	Brain - Motor cortex	NA	Direct	Post-mortem	chr4 | - | 148260031-148260043	NR3C2-202	ENST00000344721	CDS	↓
NR3C2	hsa-miR-16-5p	Brain - Cingulate gyrus	NA	Direct	Post-mortem	chr4 | - | 148114119-148114131	NR3C2-202	ENST00000344721	CDS	↓
NR3C2	hsa-miR-34a-5p	Brain - Cingulate gyrus	NA	Direct	Post-mortem	chr4 | - | 148114127-148114139	NR3C2-202	ENST00000344721	CDS	↓
NR3C2	hsa-miR-34a-5p	Brain - Motor cortex	NA	Direct	Post-mortem	chr4 | - | 148080768-148080780	NR3C2-202	ENST00000344721	3UTR	↓
NR3C2	hsa-miR-34a-5p	Brain - Motor cortex	NA	Direct	Post-mortem	chr4 | - | 148081392-148081404	NR3C2-202	ENST00000344721	CDS	↓
NR3C2	hsa-miR-34a-5p	Brain - Motor cortex	NA	Direct	Post-mortem	chr4 | - | 148154675-148154687	NR3C2-202	ENST00000344721	CDS	↓
NR3C2	hsa-miR-34a-5p	Brain - Motor cortex	NA	Direct	Post-mortem	chr4 | - | 148435825-148435837	NR3C2-202	ENST00000344721	CDS	↓
BDNF	hsa-miR-124-3p	Brain - Motor cortex	NA	Direct	Post-mortem	chr11 | - | 27658260-27658272	BDNF-201	ENST00000314915	CDS	↓
DRD4	hsa-miR-16-5p	Brain - Primary	NA	Direct	Post-mortem	chr11 | + | 639444-639456	DRD4-201	ENST00000176183	CDS	↓

Supplementary Table S1: Key data of experimentally validated miRNA/target interactions yielded from the bioinformatic analysis										
**Gene name**	**miRNA name**	**Tissue**	**Cell line**	**Experimental type**	**Phenotype**	**Binding site**	**Transcript name**	**Transcript id**	**Transcript location**	**Regulation**
		Motor Cortex								
OXTR	hsa-miR-34a-5p	Brain - Cingulate gyrus	NA	Direct	Post-mortem	chr3 | - | 8767533-8767545	OXTR-201	ENST00000316793	CDS	↓
JUN	hsa-miR-34a-5p	Brain - Cingulate gyrus	NA	Direct	Post-mortem	chr1 | - | 58781777-58781789	JUN-202	ENST00000678696	3UTR	↓
JUN	hsa-miR-34a-5p	Brain - Cingulate gyrus	NA	Direct	Post-mortem	chr1 | - | 58782176-58782188	JUN-202	ENST00000678696	CDS	↓
JUN	hsa-miR-34a-5p	Brain - Cingulate gyrus	NA	Direct	Post-mortem	chr1 | - | 58782272-58782284	JUN-202	ENST00000678696	CDS	↓
JUN	hsa-miR-34a-5p	Brain - Cingulate gyrus	NA	Direct	Post-mortem	chr1 | - | 58782687-58782699	JUN-202	ENST00000678696	CDS	↓
JUN	hsa-miR-34a-5p	Brain - Motor cortex	NA	Direct	Post-mortem	chr1 | - | 58782176-58782188	JUN-202	ENST00000678696	CDS	↓
JUN	hsa-miR-34a-5p	Brain - Motor cortex	NA	Direct	Post-mortem	chr1 | - | 58782272-58782284	JUN-202	ENST00000678696	CDS	↓
JUN	hsa-miR-34a-5p	Brain - Primary Visual Cortex	NA	Direct	Post-mortem	chr1 | - | 58782176-58782188	JUN-202	ENST00000678696	CDS	↓

**Supplementary Table S1:** Key data of experimentally validated miRNA/target interactions yielded from the bioinformatic analysis										
**Gene name**	**miRNA name**	**Tissue**	**Cell line**	**Experimental type**	**Phenotype**	**Binding site**	**Transcript name**	**Transcript id**	**Transcript location**	**Regulation**
JUN	hsa-miR-124-3p	Brain - Cingulate gyrus	NA	Direct	Post-mortem	chr1 | - | 58781572-58781584	JUN-202	ENST00000678696	3UTR	↓
JUN	hsa-miR-124-3p	Brain - Motor cortex	NA	Direct	Post-mortem	chr1 | - | 58781572-58781584	JUN-202	ENST00000678696	3UTR	↓
RELA	hsa-miR-34a-5p	Brain - Cingulate gyrus	NA	Direct	Post-mortem	chr11 | - | 65661735-65661747	RELA-201	ENST00000308639	CDS	↓
RELA	hsa-miR-34a-5p	Brain - Motor cortex	NA	Direct	Post-mortem	chr11 | - | 65653858-65653870	RELA-201	ENST00000308639	3UTR	↓
RELA	hsa-miR-34a-5p	Brain - Motor cortex	NA	Direct	Post-mortem	chr11 | - | 65661735-65661747	RELA-201	ENST00000308639	CDS	↓
RELA	hsa-miR-34a-5p	Brain - Primary Visual Cortex	NA	Direct	Post-mortem	chr11 | - | 65653607-65653619	RELA-201	ENST00000308639	3UTR	↓
RELA	hsa-miR-34a-5p	Brain - Primary Visual Cortex	NA	Direct	Post-mortem	chr11 | - | 65653923-65653935	RELA-201	ENST00000308639	3UTR	↓
RELA	hsa-miR-34a-5p	Brain - Primary Visual Cortex	NA	Direct	Post-mortem	chr11 | - | 65661815-65661827	RELA-201	ENST00000308639	CDS	↓

**Supplementary Table S1:** Key data of experimentally validated miRNA/target interactions yielded from the bioinformatic analysis										
**Gene name**	**miRNA name**	**Tissue**	**Cell line**	**Experimental type**	**Phenotype**	**Binding site**	**Transcript name**	**Transcript id**	**Transcript location**	**Regulation**
RELA	hsa-miR-124-3p	Brain - Cingulate gyrus	NA	Direct	Post-mortem	chr11 | - | 65658413-65658425	RELA-201	ENST00000308639	CDS	↓
FOSL2	hsa-miR-34a-5p	Brain - Cingulate gyrus	NA	Direct	Post-mortem	chr2 | + | 28412272-28412284	FOSL2-202	ENST00000379619	CDS	↓
FOSL2	hsa-miR-34a-5p	Brain - Cingulate gyrus	NA	Direct	Post-mortem	chr2 | + | 28413407-28413419	FOSL2-202	ENST00000379619	3UTR	↓
FOSL2	hsa-miR-34a-5p	Brain - Motor cortex	NA	Direct	Post-mortem	chr2 | + | 28412280-28412292	FOSL2-202	ENST00000379619	CDS	↓
FOSL2	hsa-miR-34a-5p	Brain - Motor cortex	NA	Direct	Post-mortem	chr2 | + | 28415082-28415094	FOSL2-202	ENST00000379619	3UTR	↓
FOSL2	hsa-miR-34a-5p	Brain - Motor cortex	NA	Direct	Post-mortem	chr2 | + | 28416827-28416839	FOSL2-202	ENST00000379619	3UTR	↓
FOSL2	hsa-miR-34a-5p	Brain - Motor cortex	NA	Direct	Post-mortem	chr2 | + | 28416697-28416709	FOSL2-202	ENST00000379619	3UTR	↓
FOSL2	hsa-miR-124-3p	Brain - Motor cortex	NA	Direct	Post-mortem	chr2 | + | 28412831-28412843	FOSL2-202	ENST00000379619	3UTR	↓
FOSL2	hsa-miR-146a-5p	Brain - Cingulate gyrus	NA	Direct	Post-mortem	chr2 | + | 28415866-28415878	FOSL2-202	ENST00000379619	3UTR	↓

Supplementary Table S1: Key data of experimentally validated miRNA/target interactions yielded from the bioinformatic analysis										
**Gene name**	**miRNA name**	**Tissue**	**Cell line**	**Experimental type**	**Phenotype**	**Binding site**	**Transcript name**	**Transcript id**	**Transcript location**	**Regulation**
JUNB	hsa-miR-30a-5p	Brain - Motor cortex	NA	Direct	Post-mortem	chr19 | + | 12792306-12792318	JUNB-201	ENST00000302754	CDS	↓
JUNB	hsa-miR-30a-5p	Brain - Motor cortex	NA	Direct	Post-mortem	chr19 | + | 12792977-12792989	JUNB-201	ENST00000302754	3UTR	↓
JUNB	hsa-miR-34a-5p	Brain - Cingulate gyrus	NA	Direct	Post-mortem	chr19 | + | 12792363-12792375	JUNB-201	ENST00000302754	CDS	↓
JUNB	hsa-miR-34a-5p	Brain - Motor cortex	NA	Direct	Post-mortem	chr19 | + | 12792363-12792375	JUNB-201	ENST00000302754	CDS	↓
IRF2	hsa-miR-34a-5p	Brain - Motor cortex	NA	Direct	Post-mortem	chr4 | - | 184429010-184429022	IRF2-210	ENST00000696840	CDS	↓
JUND	hsa-miR-34a-5p	Brain - Cingulate gyrus	NA	Direct	Post-mortem	chr19 | - | 18280833-18280845	JUND-201	ENST00000252818	CDS	↓
JUND	hsa-miR-34a-5p	Brain - Cingulate gyrus	NA	Direct	Post-mortem	chr19 | - | 18281131-18281143	JUND-201	ENST00000252818	CDS	↓
JUND	hsa-miR-34a-5p	Brain - Primary Motor Cortex	NA	Direct	Post-mortem	chr19 | - | 18280195-18280207	JUND-201	ENST00000252818	3UTR	↓
JUND	hsa-miR-30a-5p	Brain - Primary	NA	Direct	Post-mortem	chr19 | - | 18279779-18279791	JUND-201	ENST00000252818	3UTR	↓

**Supplementary Table S1:** Key data of experimentally validated miRNA/target interactions yielded from the bioinformatic analysis										
**Gene name**	**miRNA name**	**Tissue**	**Cell line**	**Experimental type**	**Phenotype**	**Binding site**	**Transcript name**	**Transcript id**	**Transcript location**	**Regulation**
		Motor Cortex								
JUND	hsa-miR-146a-5p	Brain - Primary Motor Cortex	NA	Direct	Post-mortem	chr19 | - | 18279779-18279791	JUND-201	ENST00000252818	3UTR	↓
PTGS2	hsa-miR-34a-5p	Brain - Cingulate gyrus	NA	Direct	Post-mortem	chr1 | - | 186674523-186674535	PTGS2-205	ENST00000680451	CDS	↓
PTGS2	hsa-miR-34a-5p	Brain - Cingulate gyrus	NA	Direct	Post-mortem	chr1 | - | 186676666-186676678	PTGS2-205	ENST00000680451	CDS	↓
PTGS2	hsa-miR-34a-5p	Brain - Motor cortex	NA	Direct	Post-mortem	chr1 | - | 186672624-186672636	PTGS2-205	ENST00000680451	3UTR	↓
PTGS2	hsa-miR-34a-5p	Brain - Primary Visual Cortex	NA	Direct	Post-mortem	chr1 | - | 186672624-186672636	PTGS2-205	ENST00000680451	3UTR	↓
PTGS2	hsa-miR-30a-5p	Brain - Motor cortex	NA	Direct	Post-mortem	chr1 | - | 186677733-186677745	PTGS2-205	ENST00000680451	CDS	↓
MX1	hsa-miR-34a-5p	Brain - Cingulate gyrus	NA	Direct	Post-mortem	chr21 | + | 41446021-4144603	MX1-217	ENST00000679386	CDS	↓

**Supplementary Table S1:** Key data of experimentally validated miRNA/target interactions yielded from the bioinformatic analysis										
**Gene name**	**miRNA name**	**Tissue**	**Cell line**	**Experimental type**	**Phenotype**	**Binding site**	**Transcript name**	**Transcript id**	**Transcript location**	**Regulation**
MX1	hsa-miR-34a-5p	Brain - Motor cortex	NA	Direct	Post-mortem	chr21 | + | 41435932-41435944	MX1-217	ENST00000679386	CDS	↓
MX1	hsa-miR-34a-5p	Brain - Motor cortex	NA	Direct	Post-mortem	chr21 | + | 41441853-41441865	MX1-217	ENST00000679386	CDS	↓
MX1	hsa-miR-34a-5p	Brain - Motor cortex	NA	Direct	Post-mortem	chr21 | + | 41449189-41449201	MX1-217	ENST00000679386	CDS	↓
MX1	hsa-miR-34a-5p	Brain - Motor cortex	NA	Direct	Post-mortem	chr21 | + | 41452700-41452712	MX1-217	ENST00000679386	CDS	↓
MX1	hsa-miR-30a-5p	Brain - Motor cortex	NA	Direct	Post-mortem	chr21 | + | 41446071-41446083	MX1-217	ENST00000679386	CDS	↓
OAS2	hsa-miR-34a-5p	Brain - Cingulate gyrus	NA	Direct	Post-mortem	chr12 | + | 112987068-112987080	OAS2-201	ENST00000342315	CDS	↓
OAS2	hsa-miR-34a-5p	Brain - Motor cortex	NA	Direct	Post-mortem	chr12 | + | 112995352-112995364	OAS2-201	ENST00000342315	CDS	↓
OAS2	hsa-miR-30a-5p	Brain - Motor cortex	NA	Direct	Post-mortem	chr12 | + | 112995369-112995381	OAS2-201	ENST00000342315	CDS	↓
IFI16	hsa-miR-34a-5p	Brain - Cingulate gyrus	NA	Direct	Post-mortem	chr1 | + | 159016626-159016638	IFI16-203	ENST00000368131	CDS	↓

Supplementary Table S1: Key data of experimentally validated miRNA/target interactions yielded from the bioinformatic analysis

Gene name	miRNA name	Tissue	Cell line	Experimental type	Phenotype	Binding site	Transcript name	Transcript id	Transcript location	Regulation
IFI16	hsa-miR-34a-5p	Brain - Primary Visual Cortex	NA	Direct	Post-mortem	chr1 | + | 159053605-159053617	IFI16-203	ENST00000368131	CDS	↓
FOSB	hsa-miR-34a-5p	Brain - Motor cortex	NA	Direct	Post-mortem	chr19 | + | 45472754-45472766	FOSB-210	ENST00000591858	CDS	↓
IRF7	hsa-miR-34a-5p	Brain - Motor cortex	NA	Direct	Post-mortem	chr11 | - | 614360-614372	IRF7-206	ENST00000525445	CDS	↓
MX2	hsa-miR-34a-5p	Brain - Cingulate gyrus	NA	Direct	Post-mortem	chr21 | + | 41377944-41377956	MX2-201	ENST00000330714	CDS	↓
PTGS1	hsa-miR-34a-5p	Brain - Cingulate gyrus	NA	Direct	Post-mortem	chr9 | + | 122393196-122393208	PTGS1-202	ENST00000362012	3UTR	↓
PTGS1	hsa-miR-34a-5p	Brain - Motor cortex	NA	Direct	Post-mortem	chr9 | + | 122395224-122395236	PTGS1-202	ENST00000362012	3UTR	↓
FOS	hsa-miR-30a-5p	Brain - Motor cortex	NA	Direct	Post-mortem	chr14 | + | 75282172-75282184	FOS-201	ENST00000303562	3UTR	↓
IFI16	hsa-miR-16-5p	Brain - Cingulate gyrus	NA	Direct	Post-mortem	chr1 | + | 159055010-159055022	IFI16-203	ENST00000368131	3UTR	↓

**Supplementary Table S1:** Key data of experimentally validated miRNA/target interactions yielded from the bioinformatic analysis										
**Gene name**	**miRNA name**	**Tissue**	**Cell line**	**Experimental type**	**Phenotype**	**Binding site**	**Transcript name**	**Transcript id**	**Transcript location**	**Regulation**
IFIT1	hsa-miR-124-3p	Brain - Motor cortex	NA	Direct	Post-mortem	chr10 | + | 89403038-89403050	IFIT1-202	ENST00000546318	CDS	↓
IFIT1	hsa-miR-30a-5p	Brain - Primary Visual Cortex	NA	Direct	Post-mortem	chr10 | + | 89405087-89405099	IFIT1-202	ENST00000546318	3UTR	↓
IL1A	hsa-miR-30a-5p	Brain - Motor cortex	NA	Direct	Post-mortem	chr2 | - | 112774931-112774943	IL1A-201	ENST00000263339	3UTR	↓
IFI27	hsa-miR-34a-5p	Brain - Motor cortex	NA	Direct	Post-mortem	chr14 | + | 94115822-94115834	IFI27-206	ENST00000614204	CDS	↓
IFI27	hsa-miR-34a-5p	Brain - Motor cortex	NA	Direct	Post-mortem	chr14 | + | 94115822-94115834	IFI27-206	ENST00000614204	CDS	↓
IL1B	hsa-miR-34a-5p	Prostate epithelial cells	PC3	Direct	Malignant	chr2 | - | 112832753-112832765	IL1B-201	ENST00000263341	CDS	↓
